# Bronchiolitis guidelines: what about the Italian situation in a primary care setting?

**DOI:** 10.1186/s13052-023-01527-3

**Published:** 2023-09-19

**Authors:** Federica Porcaro, Renato Cutrera, Anna Chiara Vittucci, Alberto Villani

**Affiliations:** 1 Pediatric Pulmonology and Cystic Fibrosis Unit, Bambino Gesù Children’s Hospital, IRCCS, piazza di Sant’Onofrio 4, Rome, 00165 Italy; 2 Pediatric Unit, Pediatric Emergency Department, Bambino Gesù Children’s Hospital, IRCCS, Rome, Italy; 3 Department of Systems Medicine, University of Rome “Tor Vergata”, Rome, Italy

**Keywords:** Acute viral bronchiolitis, Guidelines, Management, Outpatient setting

## Abstract

Acute viral bronchiolitis is the most common cause of hospitalization in children under 12 months of age. The variable clinical presentation and the potential for sudden deterioration of the clinical conditions require a close monitoring by healthcare professionals.

In Italy, first access care for children is provided by primary care physicians (PCPs) who often must face to a heterogeneous disease presentation that, in some cases, make the management of patient with bronchiolitis challenging. Consequently, Italian studies report poor adherence to national and international guidelines processed to guide the clinicians in decision making in acute viral bronchiolitis.

This paper aims to identify the potential factors contributing to the lack of adherence to the suggested guidelines derived by clear and evidence-based recommendations among primary care physicians operating in an outpatient setting, with a specific focus on the context of Italy. Particularly, we focus on the prescription of medications such as β2-agonists, systemic steroids, and antibiotics which are commonly prescribed by PCPs to address conditions that can mimic bronchiolitis.

**Supplementary Information:**

The online version contains supplementary material available at 10.1186/s13052-023-01527-3.

## Introduction

Acute viral bronchiolitis is the leading cause of hospital admission for lower respiratory tract infections in infant aged < 1 year [1]. The term “bronchiolitis” usually refers to the first viral episode of wheezing in infants. The age to which the definition refers is variable, considering children younger than 12 months in Europe [2–5] and Australasia [6], and younger than 24 months in United States [7], Canada [8], Spain [9] and South Africa [10].

Respiratory Syncytial Virus (RSV) is primarily associated with bronchiolitis, especially in infants aged < 6 months, although other viruses can be involved in older patients (Rhinovirus, human Bocavirus, Metapneumovirus, Parainfluenza virus, Influenza virus, Adenovirus, Coronavirus) [11]. Viral co-infections are obviously possible as bacterial ones with *Bordetella pertussis* and atypical bacteria (*Mycoplasma* as well as *Chlamydia pneumoniae)*, and in these cases the clinical course may be more severe [12, 13].

The incubation period ranges between 4 and 6 days, after which signs of upper respiratory tract infection occur. Subsequently, lower respiratory tract involvement becomes evident with variable degree of breathing difficulty, crackles and bilateral wheezing upon chest examination [12].

Despite the advancement in knowledge of the inflammatory response induced by viral agents, the underlying mechanisms are not fully understood [11]. In any case, inflammation is characterized by cellular infiltration of the peribronchiolar tissue, oedema of the bronchioles, mucus overproduction, and inefficient mucous clearance. All together, these conditions contribute to variable degree of airway obstruction, bronchospasm and air trapping, which are more severe in older children (> 6 months) with atopic predisposition and infected by Rhinovirus (RV) [11].

The unpredictable nature of the inflammatory response makes viral bronchiolitis a quite dynamic disease [11]. The sudden changes in clinical findings and possible worsening around 3–5 days after symptom onset require a close monitoring that can be challenging to apply in a primary care setting, thus also limiting the therapeutic approach.

For this reason, we evaluated the existing guidelines on acute viral bronchiolitis, focusing on the medical therapies (inhaler β2-agonists, systemic steroids, antibiotics) commonly used in the treatment of viral wheezing or asthma.

A search was conducted on Pubmed for papers with keywords as “acute AND viral AND bronchiolitis AND guidelines”, which yielded 54 results; additionally, a search using the keywords “bronchiolitis AND recommendations” resulted in 561 results. Only manuscripts that met the criteria of guidelines and published in the last 15 years were included. A total of seven relevant documents were selected. Furthermore, we decided to include the 2015 NICE UK guidelines on bronchiolitis management, even though they were not found in the electronic motor search, in order to provide insight into the British context as well.

This process of analysis was made to assess the real applicability of national guidelines in a context different from the hospital setting.

## The huge sea of bronchiolitis guidelines

To face the intrinsic dynamism of acute bronchiolitis, several guidelines have been published to assist clinicians in its diagnosis and treatment [2–10].

The most well-known document, drawn up by the American Academy of Pediatrics (AAP) [7], has served as a source of inspiration for other national societies [2, 4–6, 8], although three documents dated back to an earlier period its publication [3, 9, 10].

**Fig. 1:**
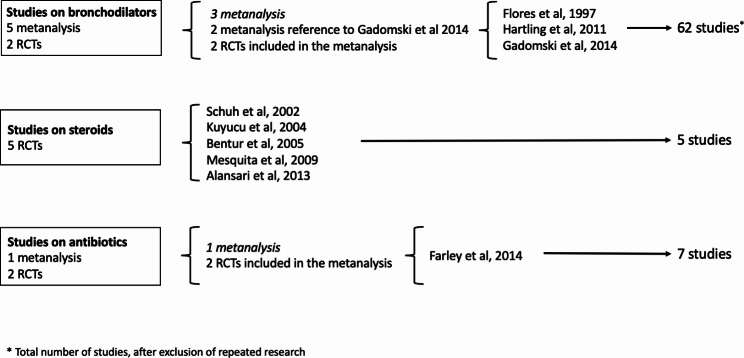
Graph representing the main studies considered by the 2014 AAP guidelines about the use of bronchodilators, steroids, and antibiotics in acute viral bronchiolitis

The 2014 AAP recommendations derive fundamentally from the conclusions of three metanalysis [14–16], five randomized controlled trials (RCTs) [17–21] and one metanalysis [22], respectively for bronchodilators, steroids and antibiotics’ use (Fig. 1).

All the available national and international documents on bronchiolitis management agree in recommending a deimplementation of unnecessary therapies, suggesting a minimal therapeutic approach consisting in gentle cleaning of the upper airway, proper child’s hydration and oxygenation [3–10].

The use of inhaler bronchodilators is widely discouraged like summarized in Table 1. Some exceptions are represented by the 2010 South Africa guidelines that considered the possibility of a trial with nebulized β2-agonists in hypoxic infants [10], and by the French guidelines [3] and the 2014 Italian guidelines [2] that considered the possibility of a trial with nebulized β2-agonists in patients with recurrent wheeze, atopic history, and unclear clinical features. However, the last update of the Italian document excludes any use of inhaler bronchodilators in bronchiolitis management, aligning with what previously suggested by the AAP [7], and most of the documents included in the systematic review of Kirolos et al [23]. This position may have been influenced by the information note issued by the Italian Medicine Agency (AIFA) on October 27, 2014. The note restricted the use of salbutamol drops (5 mg/ml) to children aged 2 years and older. However, it is important to note that this restriction was implemented specifically in response to an escalation in reported adverse reactions caused by dosing errors, incorrect administration route, or drug exchange among patients aged under 2 years. It did not extend to other formulations such as salbutamol spray administered with a spacer or the combination with ipratropium bromide [24].

**Table 1:** International guidelines on bronchiolitis: focus on recommendations about inhaler β2-agonists, systemic steroids and antibiotics use

	South Africa 2010 [10]	Spain 2010 [9]	France 2013 [3]	AAP (USA) 2014 [7]	CPS (Canada) 2014 [8]	NICE (UK) 2015 [4]	Australia NZ 2019 [6]	Italy 2022 [5]
**Child’s age**	< 24 m	< 24 m	< 12 m	1–23 m	< 24 m	n.s.	< 12 m	< 12 m
**β2-agonists**	n.r.*	n.r.	n.r.°	n.r.	n.r.	n.r.	n.r.	n.r.
**Steroids**	n.r.	n.r.	n.r.	n.r.	n.r.	n.r.	n.r.	n.r.
**Antibiotics**	n.s.	n.r.•	n.r.•	n.r.•	n.r.•	n.r.	n.r.	n.r.•

Legend: m; months; n.s., not specified; n.r., not recommended

* Trials with β2-agonists can be considered in hypoxic infants

° Trial with β2-agonists can be considered in child with recurrent wheeze, atopic history, and not clear clinical features

• Antibiotic use is allowed in presence of documented secondary bacterial infection

As for bronchodilators, national and international guidelines do not recommend the prescription of systemic or inhaled corticosteroids for managing bronchiolitis [2–10] as the evidence showed that their use did not decrease the incidence or duration of hospitalization, neither improve the short- and long-term prognosis [25].

Reasonably, the use of systemic antibiotics is not recommended considering the viral etiology of the bronchiolitis disease, though it is suggested in presence of documented secondary bacterial infection by most of documents [2, 3, 5, 7–9], except for NICE and Australasia guidelines [4, 6].

Unfortunately, this approach risks excluding some patients who could benefit from the use of inhaler β2-agonists and systemic steroids like (1) patients where viral agents (RV or others) activate a Th2 inflammatory response, (2) patients with atopic predisposition, or (3) patients with moderate-severe clinical presentation [21, 26, 27].

Despite the aforementioned recommendations, many studies indicate a general poor adherence to the proposed guidelines among both hospital and family pediatricians [28–33].

In the latter case, the reason is likely due to the limited amount of research carried out in outpatient setting. Indeed, out of the 74 studies considered by the 2014 AAP guidelines, only 7 were completed in an outpatient or outpatient/emergency department [17, 19, 34–38] after excluding repeated research.

This failure to comply with the suggested recommendations is likewise reported in the primary care setting of the Italian healthcare system.

## Limitations in applicability of the national bronchiolitis guidelines in outpatient setting

The origin of the poor compliance to proposed recommendations is probably multifactorial, mainly considering the heterogeneity of the studies included in the documents. This heterogeneity makes clinical interpretation and, consequently, decision treatment about the prescription of β2-agonists, steroids and antibiotics challenging especially within an outpatient setting.

**Fig. 2:**
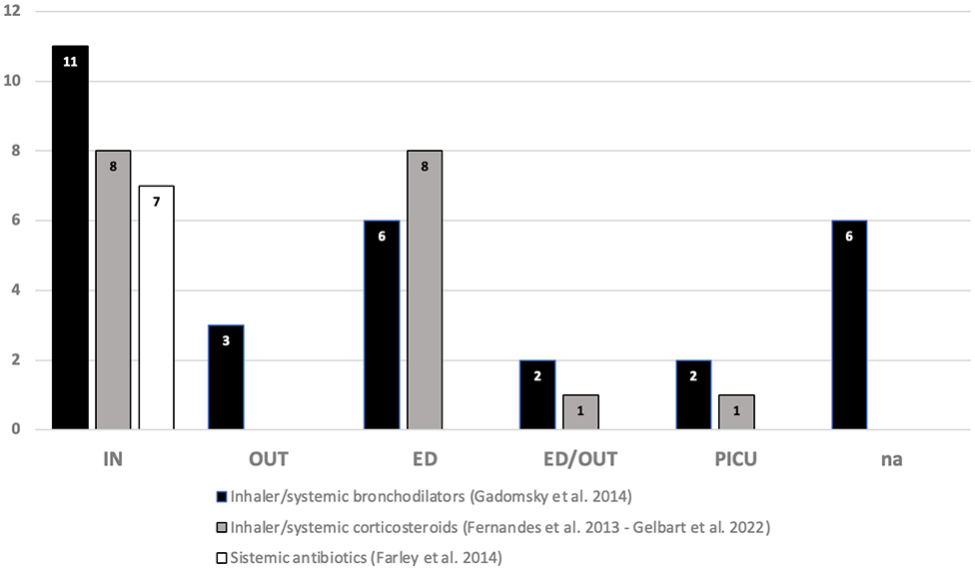
Research setting within which the studies have been carried out

Indeed, the review of the RCTs – most of which examined by the included metanalysis [15, 22, 39] – highlighted the insufficient number of studies carried out in *outpatient setting* (Fig. 2).

The metanalysis of Gadomsky [15] looking at 30 RCTs about the efficacy and safety of inhaler or systemic bronchodilators, included only 3 studies carried out in outpatient setting [36, 37, 40] and 2 studied carried out in a mixed setting (emergency department/outpatient) [34, 35].

The first outpatient study by Gupta et al. [36] examined 140 children under 12 months with the first episode of wheezing and evidence of viral respiratory tract infection. Enrolled patients had mild disease and virus typing detected both RSV and other viruses. Oral salbutamol was administered as the active treatment to 70 patients, while the remaining 70 received a placebo. According to the defined primary outcome, oral salbutamol seemed to be not superior to placebo in reducing the duration of symptoms in mild acute bronchiolitis. In Italy the use of oral salbutamol has been abandoned for decades.

The second study by Alario et al. [37] examined 74 children aged < 36 months with unspecified acute wheezing. In this study as well, mild disease was caused by RSV or other viruses. The active treatment was nebulized metaproterenol sulfate. Variation in heart rate, respiratory rate, and oxygen saturation represented the primary outcomes. The Authors concluded that nebulized metaproterenol sulfate effectively relieved respiratory distress in children with acute wheezing due to viral infection.

Lastly, the third study by Schuh et al. [40] included 40 children aged < 24 months with the first episode of wheezing and evidence of viral respiratory infection caused by RSV and others viruses. In this case, Authors didn’t specify the severity disease and nebulized albuterol was the active treatment. The therapy was randomly administered to 20 children compared to placebo. The study concluded that nebulized albuterol was a safe and effective treatment leading to improvements in accessory muscle score, respiratory rate and O2 saturation in infants with viral bronchiolitis.

The main limitation of these three studies is the evident heterogeneity in their design, including simple size, age range, disease severity, bronchiolitis definition, active treatment and its administration route, and primary outcomes. As a result, the conclusions cannot be widely applied to an outpatient setting.

However, it should be noted that positive effects of inhaled β2-therapy were found in two studies and the non-utility of oral salbutamol was confirmed.

Regarding the eighteen-research focusing on the use of inhaler or systemic steroids in acute bronchiolitis [39, 41], only one study was conducted in a mixed setting (emergency department/outpatient) [19].

Furthermore, none of the seven studied examined by Farley and colleagues on the usefulness of antibiotics in viral bronchiolitis was carried out in an outpatient setting [22]. The lack of studies investigating the efficacy of antibiotics in non-hospital setting is understandable. It is justifiable that conducting a study on the use of antibiotics in a condition primarily caused by viruses, particularly in cases with uncomplicated clinical presentations where bacterial coinfection is ruled out, would not be warranted.

It results that recommendations are primarily based on research that does not consistently represent the spectrum of patients evaluated in the primary care setting.

Additionally, when we focus on the studies carried out in outpatient setting, the aforementioned heterogeneity affects other issues briefly discussed below, and also summarized in Table 2.

**Table 2:** Synopsis of randomized control trials carried out in outpatient or outpatient/ED setting evaluated by metanalysis included in the updated version of the Italian bronchiolitis guidelines 2022

Study	Patients’ n.	Patients’ age	Setting	Viral typing	Severity	Active treatment	Outcomes
Gupta, *2008* [36]	140	< 12 m	OUT	RSV & O	Mild	Oral salbutamol	Time to illness resolution
Schuh, *1990* [40]	40	< 24 m	OUT	RSV & O	n.a.	Nebulized albuterol	Improvement in accessory muscle score, RR and O2 saturation
Alario, *1992* [37]	74	< 36 m	OUT	RSV & O	Mild	Nebulized metaproterenol sulfate	Variation of HR, RR, O2 saturation
Gadomski, *1994* [34]	88	< 12 m	OUT/ED	RSV & O	n.a.	Nebulized and oral albuterol	Variation of RR and HR, clinical score, O2 saturation (T0’, T30’, T60’)
Gadomski, *1994* [35]	128	< 12 m	OUT/ED	RSV & O	n.a.	Nebulized and oral albuterol	Variation of RR and HR, clinical score, and O2 saturation
Kuyucu, *2004* [19]	69	< 24 m	OUT/ED	n.a.	Mild-moderate	Intramuscular dexametasone	Variation of HR, RDAI score (T30’, T60’, T90’, T120’; 24 h and 5 days)

Legend: m, months; OUT, outpatient; OUT/ED, outpatient/emergency department; RSV & O, respiratory syncytial virus and others; n.a., not available; RR, respiratory rate, HR, heart rate; T0’, 30’, 60’, 90’, 120’, evaluation respectively at baseline, 30 min, 60 min, 90 and 120 min after the administration therapy; h, hours

Firstly, there is a lack of consistency in the *definition of bronchiolitis* that is sometimes not defined [34], or variably considered as nonspecific acute wheezing [19, 37], or as the first episode of acute wheezing with clinical evidence of viral respiratory tract infection [35, 36, 40].

Studies also differ in the age range of disease onset, including children aged under 36 [37], 24 [19, 40] or 12 months [34–36]. The lack of homogeneity in the age of the included patients is surprising, given that the age at which the disease presents is an important factor that should not be overlooked. It is noteworthy that among the two predominant viruses causing bronchiolitis, RSV is commonly detected in children below one year of age, with approximately 80% of cases occurring in children under three months. On the other hands, RV is more frequently found in children over the age of one year [26].

The reason of poor definition of bronchiolitis is likely due to the multifaceted variety of its clinical presentation, which depends on the age at infection, triggering factors, and genetic background influencing the inflammatory response. All three of these factors may contribute to reversible airway obstruction responsive to inhaler bronchodilators in a specific subset of children with bronchiolitis.

Unfortunately, there is no way to predict which patient will respond without a trial of therapy.

In these cases, the *viral typing* could guide the therapeutic choice. Indeed, Rhinovirus and other viruses are most often the causes of wheezing responders to some of the therapies recommended against by the available guidelines [11]. However, the studies carried out in outpatient setting do not always specify the viral typing [19] or include both patients with RSV and other viral infections [34–37, 40]. On this point too, the research is poor representative of the Italian primary care setting where the viral typing is challenging to apply and the clinical picture of the infant < 2 years with the first episode of wheezing and initially evaluated by general pediatrician remains undifferentiated, both in reference to the etiology and response to drugs (β2-agonists and steroids). Indeed, it cannot be ruled out that some children with other underlying conditions or who will develop asthma later in the life, may experience their first episode of wheezing in the age range of bronchiolitis, thus making the differential diagnosis more difficult (Table 3).

**Table 3:** Conditions to be consider in the differential diagnosis of infant (< 12 months) with first episode of wheezing in outpatient setting

Differential diagnosis of conditions that may cause wheezing in infants	
Acute viral bronchiolitis	
Aspiration pneumonia	
Asthma	
Bordetella pertussis infection	
Chronic pulmonary disease	
Congenital heart disease	
Foreign body aspiration	
Pneumonia	
Pulmonary artery sling	
Viral-triggered wheezing	

Therefore, the failure of PCPs to perform this differentiation process unavoidably results in poor adherence to the recommendations proposed by national and international guidelines.

Finally, another aspect to consider concerns the *severity assessment*. We know that 32 scoring instruments are available to assess bronchiolitis severity, but as reported by Rodriguez-Martinez et al., further work is needed to develop validated instruments to uniformly score the disease severity [42]. When we focus on this issue among the studies carried out in outpatient setting, severity assessment is not reported in three studies [34, 35, 40], only two studies include patients with mild disease [36, 37], and one enrolls infants with mild-moderate bronchiolitis [19]. As a result, there is an insufficient representation of the possible severity of clinical conditions that pediatricians may encounter, and in some moderate-severe case, a trial with inhaler bronchodilator and a short course of systemic steroid may reasonably be required.

### Adherence to recommendations: the current italian situation

Recently, five Italian studies showed that the deimplementation of unnecessary therapies suggested by national and international guidelines has caused concerns among pediatric care providers, and consequently the lack of adherence to the guidelines themselves.

The survey carried out by Manti and colleagues evaluated the diagnostic and therapeutic approach to bronchiolitis among Italian pediatricians: out of the 234 pediatricians who completed the questionnaire, 144 (18.8%) were family pediatricians. Based on the collected answers, the Authors concluded that the administration of non-recommended interventions occurred at a moderate-to‐high rate: indeed, inhaled β2‐agonists, systemic steroids and antibiotics were prescribed by 39.64%, 64.52%, and 4.73% of pediatricians, respectively [30].

The retrospective study proposed by Barbieri et al. described a practice variation in the management of acute bronchiolitis among Italian PCPs after the publication of national guideline in 2014 [31]. The research included the experience of 134 family pediatricians throughout Italy and assessed the therapeutic approach adopted for bronchiolitis management in a primary care setting. Although overprescribing was still prevalent, a decrease in nebulized β2-agonists’ and systemic steroids use has been reported, while almost no variation in antibiotic prescription has been recorded. According to the Authors’ considerations, this misuse found justification in the uncertainty in differentiating between bronchiolitis and bacterial pneumonia in a primary care setting.

However, the failure to follow the bronchiolitis guidelines is not only an issue that concerns the primary care setting.

Biagi et al. reported a decade-long experience in an Italian hospital, comparing the management of two groups of patients belonging respectively to the pre-guidelines’ era and the post-guidelines’ one. The comparison revealed a significant reduction in the prescription of systemic steroids (58.9% vs. 41.8%, p < 0.001) and antibiotics (59.5% vs. 42.3%, p < 0.001), while the use of inhaled salbutamol remained stable over time (39.4% vs. 37.6%, p = 0.505) [32].

Carlone et al. [33] reported retrospective data on 214 infants aged < 24 months and hospitalized with bronchiolitis in four pediatric hospitals. The study, in lines with the previous ones, highlighted the prescription of not recommended treatments such as inhaled therapy, systemic steroids and antibiotics in 79.4%, 34.6%, and 49.1% of patients, respectively.

A more recent retrospective and monocentric study conducted by Abbate et al. assessed the impact of the 2014 Italian Guidelines on the management of bronchiolitis in children hospitalized between 2010 and 2019 [43]. The study analyzed a cohort of 346 patients aged < 12 months, dividing them into two groups based on whether their admission was before or after the guideline’s publication. Whitin the study cohort, nasal swab was performed on 90.5% of the patients to detect respiratory viruses, with 63.9% of them positive to RSV. Consistent with the previously cited articles, the Authors concluded that the publication of guidelines led to a reduction in the utilization of chest x-ray, blood testing and inhaled or systemic steroids. However, there was no significant decrease observed in the prescription rate of antibiotics and inhaled beta2-agonists between the two groups.

### Strength and Achilles’ heel of guidelines

Overall, clinical practice guidelines are considered essential tools for improving the quality of care. To ensure the best standard of care, scientists relay on the use of evidence-based medicine (EBM), usually employed in the development of guidelines. This rigorous method, as defined by its creator David L. Sackett, involves “the explicit and conscientious use of the best current evidence in making decisions in the medical practice”. The underlying assumption of EBM is the extrapolation of research results conducted on a large patient population to the individual case.

While it is reasonable to assume that findings derived from extensive studies may be more representative of individual case, it is important to consider the possible spectrum by which a disease can manifest itself, exactly such as in the case of acute viral bronchiolitis [44]. Consequently, clinical practice can’t solely rely on the straightforward application of general results to an individual patient; it should instead be the perfect balance between the best available evidence, clinical experience and peculiar patient needs.

These speculations support the need for revising the guidelines, embracing the possibility of a personalized medicine in the management of bronchiolitis as well.

## Conclusions

While it is widely recognized that guidelines are intended to assist clinicians and not to replace physician clinical judgment in decision making, the observations reported in the present study highlight several reasons why bronchiolitis guidelines are not consistently followed in an outpatient setting.

The outpatient studies included in the metanalysis upon which the guidelines are based appear to inadequately represent the primary care setting. The limited available research carried out in the past consists of studies that differs in methodology (including bronchiolitis definition, patient age range, virus typing, severity assessment, active treatment, possibility of co-administration drugs, primary or secondary outcomes) (Table 2).

Furthermore, the guidelines do not consider the possibility of “undifferentiated patients” for whom viral testing or therapeutic trials (both discouraged by guidelines) could help in defining the “bronchiolitis syndrome”.

Last, but not least, although it is a less valid reason, it must consider that psychologic factors and parental expectations “to do something” can also contribute to noncompliance with the guidelines.

Parents cannot be asked to witness the progress of disease without practicing any therapy. In this type of situation, the choice to take the sick baby to the emergency room becomes obligatory, nullifying the possibility of PCPs to act as a filter.

Therefore, despite our intention is not to endorse arbitrary or unproven treatments, we hope the next revision of national and international guidelines on acute bronchiolitis will consider both the practice realities of front-line clinicians and the recent evidence regarding the distinct endotypes/phenotypes of bronchiolitis, thereby allowing for the possibility of a tailored treatment.

### Electronic supplementary material

Below is the link to the electronic supplementary material.

Supplementary Material 1

## Data Availability

Not applicable.

## List of Abbreviations

PCPs: Primary care physicians
RSV: Respiratory syncytial virus
RV: Rhinovirus
AAP: American Academy of Pediatrics
RCTs: Randomized controlled trials
NICE: National Institute for Health and Care Excellence
EBM: Evidence based medicine
