# Supplementary material for: Bronchiolitis guidelines: what about the Italian situation in a primary care setting?
Source: Ital J Pediatr. 2023 Sep 19;49:123. doi: 10.1186/s13052-023-01527-3 (PMC10510229; doi:10.1186/s13052-023-01527-3)
Supplement: Supplementary file 1 — Supplementary Material 1 [file 13052_2023_1527_MOESM1_ESM.docx]

Dear Editor,

We are pleased to submit our paper entitled “*Bronchiolitis guidelines: what about home care*?”, so that it can be considered for publication by the Italian Journal of Pediatrics.

The aim of the manuscript is to identify potential weaknesses in the recent national bronchiolitis guidelines that can justify the lack of adherence to the suggested recommendations among primary care physicians (PCPs) operating in an outpatient setting. Specifically, we focus on the prescription of medications which are commonly prescribed by PCPs to address conditions that can mimic bronchiolitis.

The manuscript has not been published previously and is not under consideration elsewhere.

All authors are responsible for reported research and have approved the manuscript as submitted. They don’t have any conflict of interest to disclose.

Best regards

Federica Porcaro
